# SwimCount Harvester™ versus Density Gradient Centrifugation: A Pilot Study exploring Sperm DNA Fragmentation and Motile Sperm Recovery

**DOI:** 10.1590/S1677-5538.IBJU.2026.0327

**Published:** 2026-05-10

**Authors:** Mette Brix Jensen, Betina Boel Povlsen, Natalie Heide, Jette Lykkegaard, Maria Gabriela Mulato, Sandra Sousa, Peter Humaidan, Sandro C. Esteves

**Affiliations:** 1 University Clinic for Fertility Skive Regional Hospital Denmark University Clinic for Fertility, Skive Regional Hospital, Denmark; 2 Clínica de Andrologia e Reprodução Humana Campinas SP Brasil ANDROFERT, Clínica de Andrologia e Reprodução Humana, Campinas, SP, Brasil; 3 Aarhus University Department of Clinical Medicine Denmark Department of Clinical Medicine, Aarhus University, Denmark; 4 Universidade Estadual de Campinas Faculdade de Ciências Médicas Departamento de Cirurgia Campinas SP Brasil Departamento de Cirurgia (Disciplina de Urologia), Faculdade de Ciências Médicas, Universidade Estadual de Campinas (UNICAMP), Campinas, SP, Brasil

**Keywords:** Spermatozoa, Semen Quality Analysis, DNA fragmentation, Microfluidics

## Abstract

**Purpose:**

To compare SwimCount Harvester™, a non-centrifugal sperm selection system, with Density Gradient Centrifugation (DGC) in terms of sperm DNA fragmentation (SDF), agreement between analytical methods, and total motile sperm recovery.

**Materials and Methods:**

This pilot study evaluated semen samples processed using DGC and SwimCount Harvester™. SDF was assessed in raw and processed samples using the Sperm Chromatin Dispersion (SCD; Halosperm^®^) assay, analyzed both manually and via computer-assisted sperm analysis (SCA^®^ CASA). Agreement between manual and CASA classification (>20% threshold) was evaluated using Cohen's kappa with bootstrap confidence intervals (10,000 resamples). Total motile sperm count (TMSC) was compared between DGC and Harvester (n=36), and effect size was calculated using Cohen's d.

**Results:**

Both DGC and Harvester significantly reduced SDF compared with raw semen (p<0.001 for all comparisons), with no significant difference between techniques (CASA p=0.219; manual p=0.341). Agreement between manual and CASA assessment improved following processing, reaching perfect concordance after Harvester (κ=1.00; bootstrap 95% CI 0.54–1.00). Harvester yielded significantly higher TMSC compared with DGC (p=0.001), with a large effect size (Cohen's d=0.81).

**Conclusion:**

SwimCount Harvester™ achieves SDF reduction comparable to DGC while significantly increasing motile sperm recovery. These findings support the potential of non-centrifugal sperm selection as an alternative to conventional methods. Larger studies are required to confirm these results and determine their clinical implications.

## INTRODUCTION

Sperm preparation is a critical step in assisted reproductive technology (ART), as the selection of functionally competent spermatozoa directly influences downstream reproductive processes, including fertilization and early embryonic development (1). While conventional semen analysis remains the cornerstone of male fertility evaluation, it provides limited insight into sperm functional competence, particularly at the level of chromatin integrity (2-4). Increasing evidence indicates that sperm DNA fragmentation (SDF) is associated with impaired reproductive outcomes, including reduced fertilization potential, compromised embryo development, and increased risk of miscarriage (5-9). These considerations reinforce the need to refine sperm selection strategies beyond conventional semen parameters, with increasing emphasis on functional biomarkers such as DNA integrity in both research and clinical practice.

Density Gradient Centrifugation (DGC) has long been the standard method for sperm preparation in ART laboratories, allowing enrichment of motile spermatozoa with improved morphology and reduced contamination from seminal plasma and debris (1). However, centrifugation exposes spermatozoa to mechanical stress and may promote the generation of reactive oxygen species (ROS), which have been implicated in DNA damage and functional impairment (10-12). These limitations have prompted the development of alternative sperm selection strategies aimed at minimizing iatrogenic damage during processing (12, 13).

In this context, non-centrifugal sperm selection systems have emerged as a promising approach (12, 14, 15). These technologies rely on the intrinsic ability of spermatozoa to migrate or navigate microenvironments, thereby enabling selection based on functional competence rather than physical separation alone. SwimCount Harvester™ is a migration-based sperm selection device designed to isolate motile spermatozoa without centrifugation, potentially reducing mechanical stress and oxidative injury while enriching for sperm with improved biological quality (16).

Despite these theoretical advantages, comparative evidence between non-centrifugal systems and DGC remains limited, particularly with respect to sperm DNA fragmentation and laboratory performance metrics (13). In addition, variability between manual and computer-assisted methods for SDF assessment may influence the interpretation and reproducibility of results (17).

This pilot study was therefore designed to compare DGC and SwimCount Harvester™ with respect to SDF levels, agreement between manual and CASA-based SDF assessment, and total motile sperm recovery.

## MATERIALS AND METHODS

### Study Design and Ethical Approval

This pilot comparative study evaluated laboratory parameters following sperm preparation using Density Gradient Centrifugation and SwimCount Harvester™.

The Central Denmark Region Committees on Health Research Ethics approved the conduct of this study (File number 1-10-72-9-25).

### Participants

A total of 36 men (mean age 35.8 ± 5.1 years; BMI 26.1 ± 2.7 kg/m^2^) undergoing semen analysis as part of routine fertility evaluation were included. Thirteen of the included men had an SDF analysis made. The inclusion criterion was males attending the fertility clinic who were able to provide freshly ejaculated sperm for analysis. No clinical outcomes were evaluated in this pilot study.

### Semen Analysis

Participants were instructed to maintain a period of 2 days ejaculatory abstinence before semen collection on-site. Semen analysis was performed according to the 2021 WHO laboratory manual criteria (6th edition) using SCA^®^ CASA System (Microptic, Spain) for sperm concentration and motility (18, 19).

### Sperm DNA Fragmentation Assessment

SDF was assessed using the Sperm Chromatin Dispersion (SCD) assay (Halosperm^®^, Halotech DNA, Madrid, Spain), following previously validated methodology (20). Briefly, semen aliquots were embedded in low-melting agarose on precoated slides. Controlled acid denaturation generated single-stranded DNA at strand break sites, followed by protein removal through lysis. Spermatozoa with intact DNA developed large or medium chromatin dispersion halos, whereas those with fragmented DNA exhibited small or absent halos. Slides were dehydrated and stained using Diff-Quik.

For manual analysis, a minimum of 400 spermatozoa were scored per sample under bright-field microscopy by experienced technicians, following standardized halo classification criteria (8, 9, 20, 21). The SDF rate was calculated as the percentage of fragmented spermatozoa relative to total counted.

Slides were additional analyzed using the SCA^®^ DNA Fragmentation module (Sperm Class Analyzer, Microptic, Spain) (18). Prepared slides were examined using bright-field microscopy and a minimum number of 400 spermatozoa were automatically analyzed. Individual spermatozoa were identified and classified based on halo size surrounding the core, in accordance with the Sperm Chromatin Dispersion principle. Large and medium halos were indicative of intact DNA, whereas small or absent halos as well as degraded spermatozoa, were classified as exhibiting DNA fragmentation. Fragmentation status was determined by automated assessment of morphometric parameters, primarily halo size. Image acquisition parameters, including threshold settings, were established based on prior manual evaluation and applied consistently across samples. Up to 30 fields per sample were analyzed, with priority given to achieving the required number of evaluated spermatozoa. Like the manual assessment, the SDF rate was calculated as the percentage of fragmented spermatozoa relative to total counted.

### Quality control

Quality control included visual verification of halo morphology, ensuring absence of overlapping spermatozoa, homogeneous background, and structural integrity of the gel matrix. Only slides fulfilling predefined quality criteria were included in the final analysis.

### Sperm Processing

Density gradient centrifugation was performed using SpermGrad (Vitrolife, Göteborg, Sweden) gradient (90–45%) (Figure-1). SpermGrad 100% was diluted using IVF medium (Vitrolife, Göteborg, Sweden) to obtain 90% and 45% dilutions. Two gradient columns were subsequently prepared in Falcon tubes (BD, USA) by gently layering 1.5 mL of each solution, with the 90% fraction at the bottom. The semen sample was carefully added on the top of the density gradient columns, and the samples were centrifuged at 300g for 15 min at room temperature. After centrifugation, the pellet was washed twice with 1.5 mL IVF medium followed by centrifugation at 200g for 10 min. The supernatant was removed and the pellet resuspended in IVF medium for semen analysis in the SCA^®^ CASA System (Microptic, Spain) for concentration and motility.

**Figure 1:**
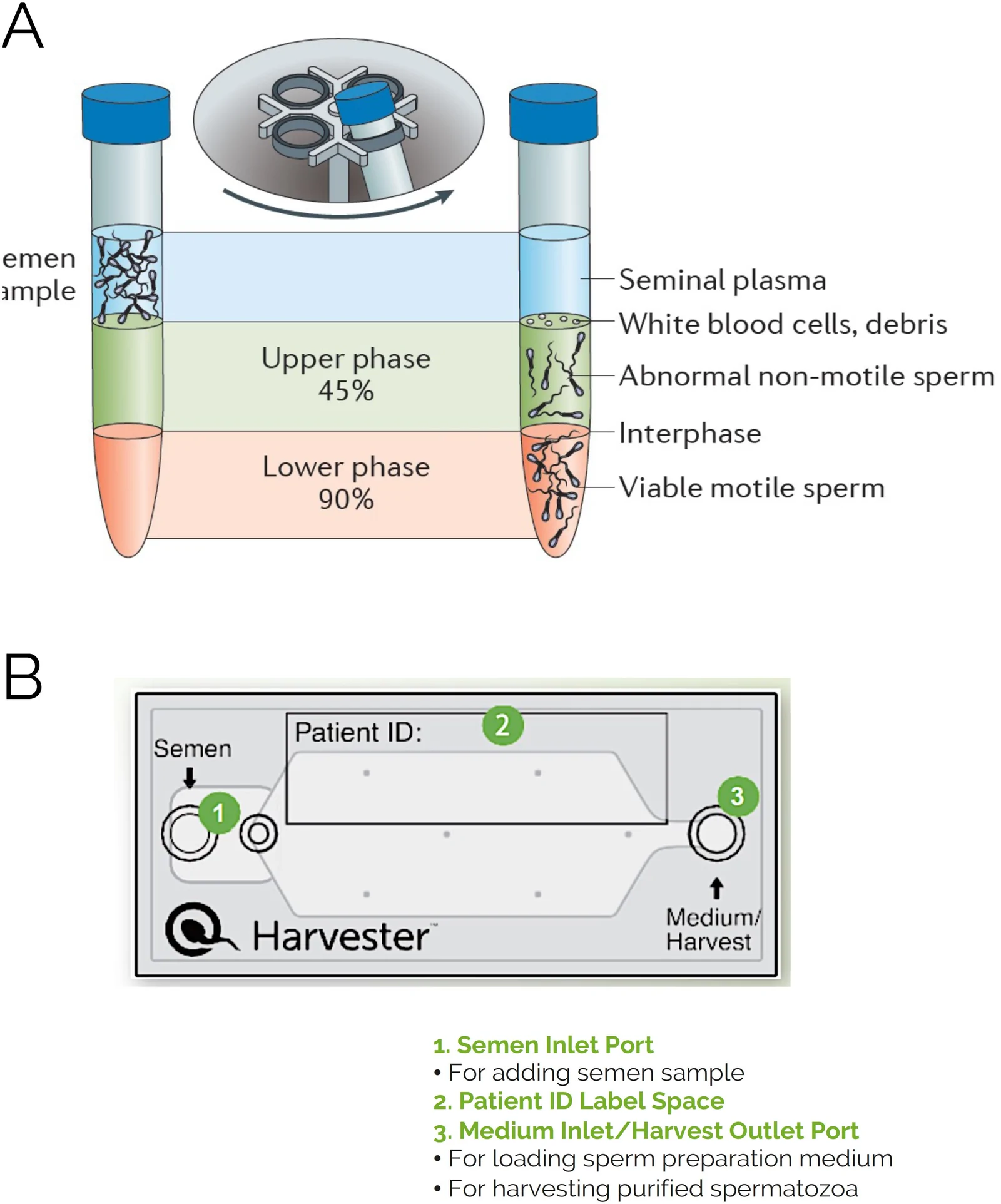
Sperm preparation techniques: Density gradient centrifugation and SwimCount Harvester™.

(A) Schematic representation of density gradient centrifugation (DGC). Semen is layered over a discontinuous gradient (45% and 90%) and centrifuged, allowing separation of spermatozoa based on density. Debris, leukocytes, and non-motile or abnormal spermatozoa remain in the upper layers, while motile, morphologically normal spermatozoa are recovered in the pellet.

(B) Schematic representation of the SwimCount Harvester™ device. Semen is introduced into the inlet chamber, and spermatozoa migrate through a membrane into the upper chamber containing culture medium. Motile spermatozoa are subsequently collected from the harvest outlet, enabling selection without centrifugation. The device includes a semen inlet port, a medium inlet/harvest outlet port, and a designated space for patient identification.

SwimCount Harvester™ preparation was performed using the SwimCount Harvester™ (MotilityCount ApS, Copenhagen, Denmark) 1 mL sperm purification device (Figure-1). The SwimCount Harvester™ has two chambers separated by a 10-μm–pore size membrane. One mL of semen was loaded into the lower chamber, and 0.8 mL of IVF medium (Vitrolife, Göteborg, Sweden) was loaded into the upper chamber. The device was incubated in a horizontal position at 37ºC for 30 minutes, after which the medium from the upper chamber, enriched in highly motile spermatozoa, was carefully collected for further analysis in the SCA^®^ CASA System (Microptic, Spain) for concentration and motility.

A schematic representation of the study design and analytical workflow is shown in Figure-2.

**Figure 2:**
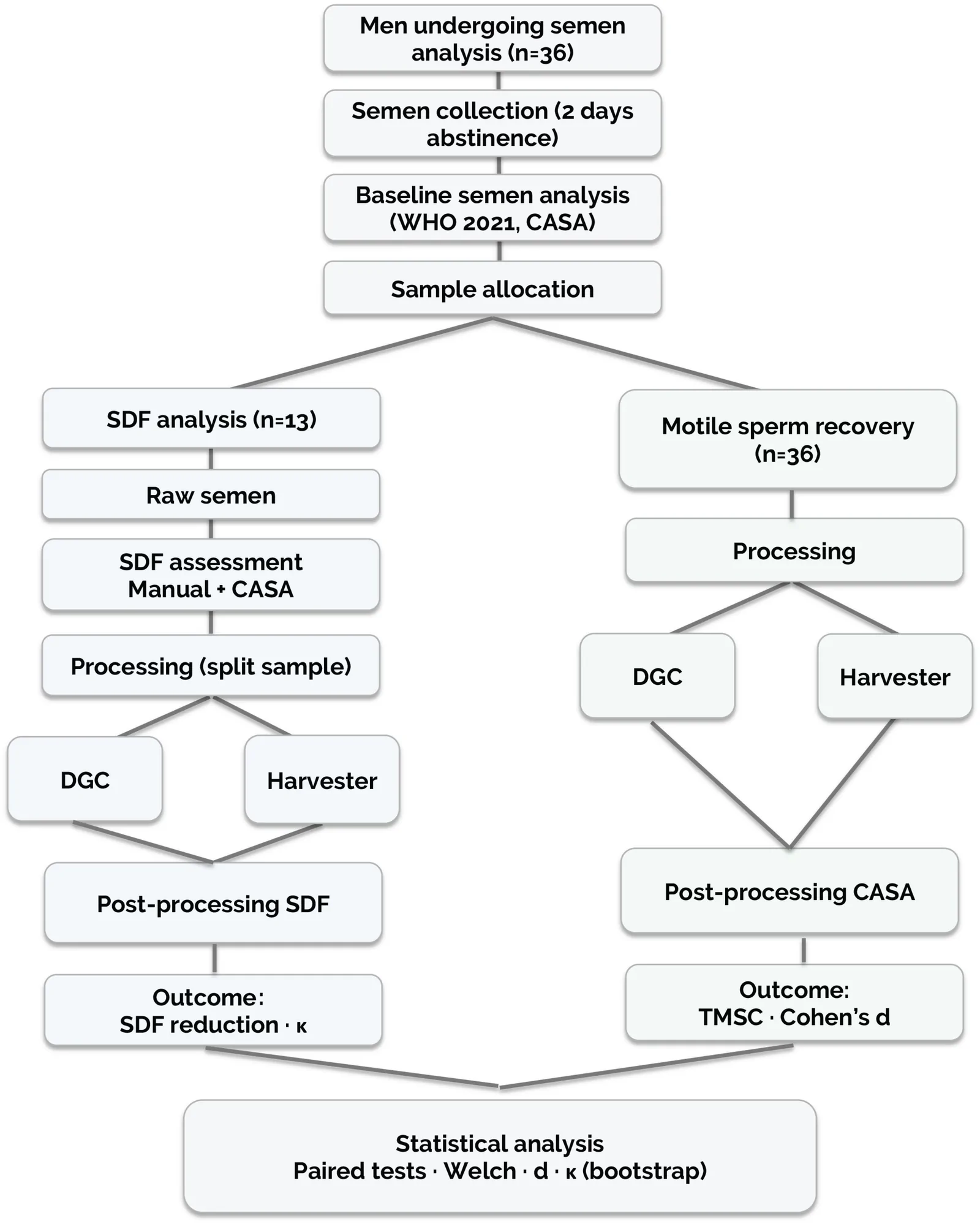
Study design and analytical workflow.

Semen samples from 36 men were collected and analyzed according to WHO 2021 criteria. A subset of samples (n = 13) underwent sperm DNA fragmentation (SDF) assessment using the sperm chromatin dispersion (SCD) assay, analyzed manually and by computer-assisted semen analysis (CASA), in raw semen and following processing by density gradient centrifugation (DGC) or SwimCount Harvester™ in a within-subject design. Total motile sperm count (TMSC) was evaluated in all samples (n = 36) following DGC and Harvester processing. Statistical analyses included paired comparisons, Cohen's d, and Cohen's κ with bootstrap confidence intervals.

### Statistical Analysis

Descriptive statistics are presented as mean, standard deviation (SD), median, minimum, maximum, and interquartile range (IQR). Normality of continuous variables was assessed using the Shapiro–Wilk test. Homogeneity of variances between independent groups was evaluated using Levene's test. Given the exploratory nature of this pilot study, no formal sample size calculation was performed.

Because SDF measurements were obtained from the same samples under different processing conditions, paired comparisons were performed when appropriate. Paired t-tests were applied when normality assumptions were satisfied. In independent comparisons with unequal variances, Welch's correction was used. Non-parametric tests were employed when distributional assumptions were not met.

Agreement between manual and CASA SDF classification (using a 20% SDF threshold to classify non-pathological versus pathological SDF results) was evaluated using Cohen's kappa coefficient. Confidence intervals were estimated using both asymptotic standard error and bias-corrected accelerated (BCa) bootstrap resampling with 10,000 iterations to improve robustness given the limited sample size.

Effect sizes for SDF reductions were calculated as standardized mean differences (Cohen's d), using pooled standard deviations. For TMSC comparisons, effect size was calculated using Cohen's d based on pooled variance. Effect sizes were interpreted according to conventional thresholds (0.2 small, 0.5 medium, 0.8 large).

Statistical significance was defined as p < 0.05. Analyses were performed using JMP software (SAS Institute, Cary, NC, USA).

## RESULTS

### Sperm DNA Fragmentation in Raw and Processed Samples

In raw semen, SDF values demonstrated considerable variability, particularly when assessed using CASA. Mean SDF measured by CASA was 24.0% (SD 15.2%), ranging from 6.0% to 58.0%, with a median of 21.8%, indicating that a considerable proportion of samples exhibited elevated DNA fragmentation. Manual SCD assessment yielded a comparable mean SDF of 25.9% (SD 9.2%) and a median of 25.0%. The narrower dispersion observed with manual assessment suggests lower variability, although this may reflect methodological differences rather than true biological variation (Table-1).

**Table 1:** Descriptive Statistics and Paired Effect Sizes of Sperm DNA Fragmentation According to Processing Method and Assessment Technique (n = 13).

Processing Method	Assessment	Mean ± SD	Median (IQR)	Min–Max	p-value vs Raw	p-value DGC vs Harvester
Raw semen	CASA	24.0 ± 15.2	21.8 (12.0–32.0)	6.0–58.0	—	—
Post-DGC	CASA	3.8 ± 4.2	3.5 (1.0–6.0)	0.0–15.1	<0.001	0.219
Post-Harvester	CASA	6.0 ± 5.0	4.3 (2.0–8.0)	0.3–14.0	<0.001	—
Raw semen	Manual SCD	25.9 ± 9.2	25.0 (20.0–32.0)	10.0–40.0	—	—
Post-DGC	Manual SCD	7.5 ± 8.4	5.0 (2.0–9.0)	0.0–25.0	<0.001	0.341
Post-Harvester	Manual SCD	6.8 ± 4.5	8.0 (3.0–10.0)	1.0–15.0	<0.001	—

CASA = computer-assisted semen analysis; SCD = sperm chromatin dispersion test; DGC = Density-gradient centrifugation; IQR = interquartile range; SD = standard deviation

Following DGC, SDF was significantly reduced. In CASA analysis, mean SDF decreased from 24.0% to 3.8% (p<0.001), corresponding to an approximate 84% relative reduction. The median value declined to 3.5%, and the interquartile range narrowed considerably, indicating enrichment of a more homogeneous sperm population (Table-1). The standardized effect size for this reduction was large (Cohen's d = 1.81). Manual SCD analysis showed a similar decrease from 25.9% to 7.5% (p<0.001), with a large effect size (d = 2.09).

SwimCount Harvester™ processing also resulted in a significant SDF reduction relative to raw semen. In CASA analysis, the mean SDF decreased to 6.0% (p<0.001), with a large effect size (d = 1.59). Manual SCD analysis showed a reduction to 6.8% (p<0.001), with an effect size of d = 2.64 (Table-1).

Direct comparison between DGC and Harvester revealed no statistically significant difference in SDF reduction (CASA p=0.219; Manual p=0.341). Although DGC showed numerically lower mean SDF values in the CASA analysis, no statistically significant difference was observed between techniques, suggesting comparable efficacy under the conditions tested (Table-1).

The reduction in SDF across processing methods is illustrated in Figure-3A.

**Figure 3:**
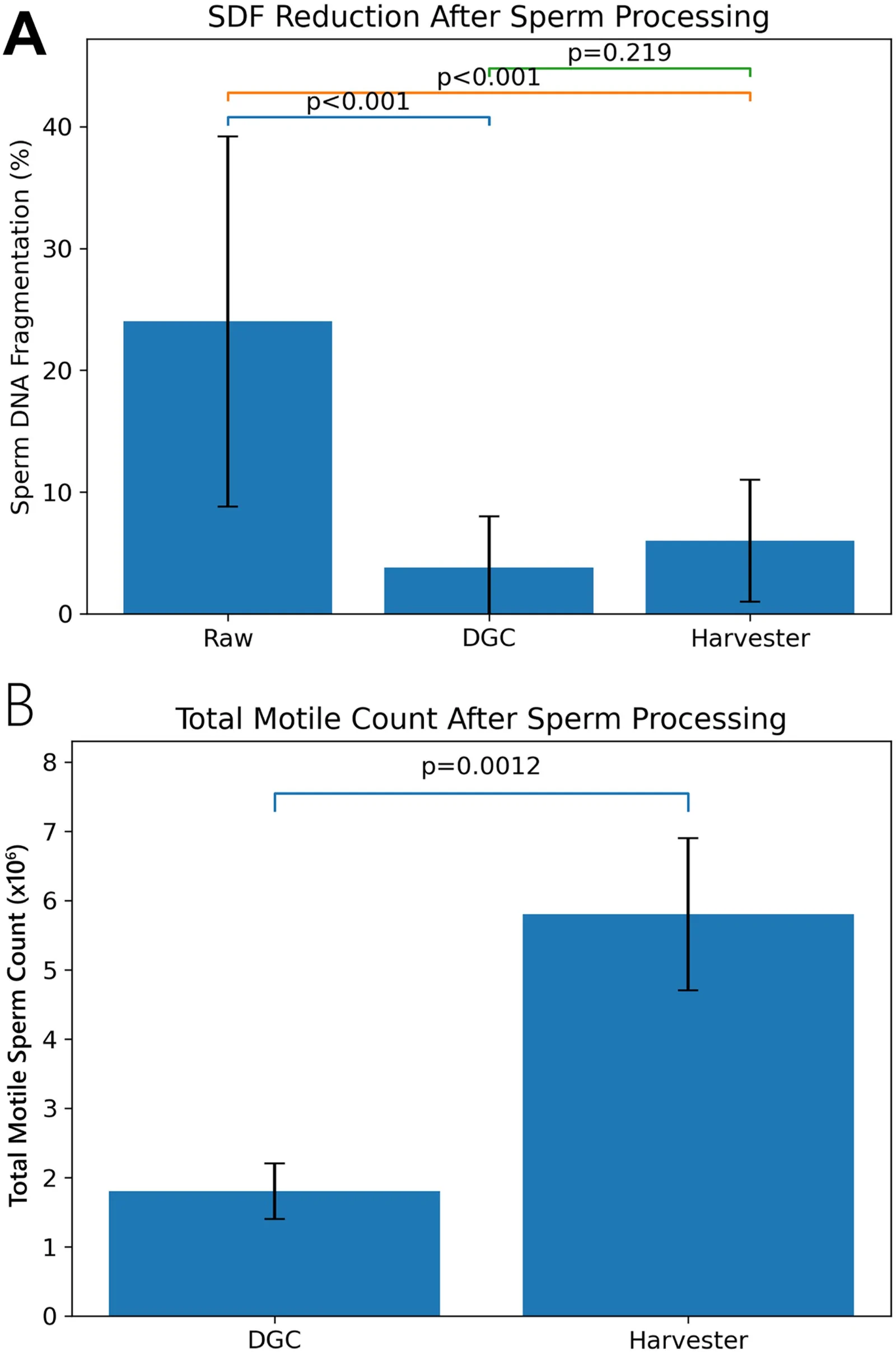
Effects of sperm processing on DNA fragmentation and motile sperm recovery.

(A) Sperm DNA fragmentation (SDF) in raw semen and following Density Gradient Centrifugation (DGC) and SwimCount Harvester™ processing. Both DGC and Harvester significantly reduced SDF compared with raw semen (p<0.001 for both comparisons). No significant difference was observed between DGC and Harvester (p=0.219).

(B) Total motile sperm count (TMC) following DGC and Harvester processing. Harvester yielded significantly higher motile sperm recovery (p=0.0012).

Data are presented as mean ± standard deviation.

### Agreement Between Manual and CASA SDF Classification

Agreement between manual SCD scoring and CASA classification improved following sperm processing. In raw semen, the observed agreement was 76.9%, corresponding to a Cohen's kappa of 0.53 (bootstrap 95% CI 0.05–0.88), indicating moderate agreement. After DGC processing, agreement increased to 84.6%, with κ = 0.66 (bootstrap 95% CI 0.18–0.93), consistent with substantial agreement. Following Harvester processing, complete concordance was observed (κ = 1.00; bootstrap 95% CI 0.54–1.00), indicating perfect agreement (Table-2).

**Table 2:** Agreement Between CASA and Manual SCD (>20% Threshold).

Processing Method	CASA+/Manual+	CASA+/Manual–	CASA–/Manual+	CASA–/Manual–	Agreement (%)	κ (Bootstrap 95% CI)
Raw semen	6	1	2	4	76.9	0.53 (0.05–0.88)
Post-DGC	3	1	1	8	84.6	0.66 (0.18–0.93)
Post-Harvester	4	0	0	9	100	1.00 (0.54–1.00)

CASA = computer-assisted semen analysis; SCD = sperm chromatin dispersion test; DGC = Density-gradient centrifugation; CI = confidence interval

### Total Motile Sperm Count

TMSC differed significantly between processing methods. DGC preparations yielded a mean TMSC of 1.8 x 10^6^ (SD 2.6), whereas Harvester preparations yielded a mean of 5.8 x 10^6^ (SD 6.5) (Table-3). Levene's test confirmed unequal variances, and Welch's correction was applied. The difference was statistically significant (p=0.001). The calculated standardized effect size was large (Cohen's d = 0.81), indicating a substantial magnitude of difference favoring Harvester in motile sperm recovery. The comparison of total motile sperm recovery between DGC and Harvester is shown in Figure-3B.

**Table 3:** Comparison of Semen Parameters following Density Gradient Centrifugation and SwimCount Harvester™ sperm selection (n=36).

	Raw semen	DGC	Harvester	p-value	Cohen's d (DGC vs Harvester)
Concentration (x10^6^/mL)	23.9 ± 18.6	3.4 ± 3.7	7.9 ± 8.4	0.004	0.69
Motility (%)	57.1 ± 18.5	89.3 ± 14.1	88.2 ± 14.0	0.74	0.08
Total sperm count (x10^6^)	67.6 ± 60.5	3.4 ± 3.7	6.3 ± 6.7	0.03	0.54
Total motile sperm count (x10^6^)	43.8 ± 44.1	1.8 ± 2.6	5.8 ± 6.5	0.001	0.81

Values are mean ± SD

## DISCUSSION

### Main findings

This pilot study provides a within-subject comparison of DGC and a non-centrifugal migration-based sperm selection system (SwimCount Harvester™) with respect to sperm DNA fragmentation (SDF), analytical agreement between manual and CASA-based assessment, and total motile sperm recovery.

Three main findings emerge. First, both DGC and Harvester significantly reduced SDF compared with raw semen, with large, standardized effect sizes. Second, no statistically significant difference in SDF reduction was observed between techniques, suggesting comparable efficacy in selecting spermatozoa with improved chromatin integrity under the conditions tested. Third, Harvester yielded significantly higher total motile sperm counts compared with DGC, with a large effect size. In addition, agreement between manual and CASA SDF assessment improved following sperm processing, reaching perfect concordance after Harvester selection. Collectively, these findings suggest that migration-based sperm selection can achieve chromatin integrity enrichment comparable to conventional centrifugation while substantially increasing motile sperm recovery.

### Interpretation

The reduction in SDF observed following both DGC and Harvester processing supports the concept that motility-based selection enriches for spermatozoa with improved chromatin integrity. This is biologically plausible, as SDF has been associated with reduced motility, oxidative stress, and impaired membrane integrity (5, 9, 15, 22, 23). By selecting motile spermatozoa, both techniques inherently reduce the proportion of fragmented cells (16, 24, 25).

The absence of a significant difference between DGC and Harvester in SDF reduction suggests that centrifugation is not essential for effective SDF reduction. Although DGC showed numerically lower SDF values in CASA-based analysis, the overlap in data dispersion and lack of statistical significance indicate comparable performance in DNA fragmentation reduction.

The improved agreement between manual and CASA-based SDF assessment following sperm processing likely reflects reduced heterogeneity in chromatin dispersion patterns (i.e., halo morphology). Raw semen samples contain a broad spectrum of halo morphologies, increasing classification variability. Following processing, the sperm population becomes more homogeneous, facilitating more consistent classification across analytical methods. The use of bootstrap confidence intervals strengthens the robustness of this observation despite the limited sample size.

The most notable laboratory difference between techniques was observed in total motile sperm recovery. Harvester yielded significantly higher TMSC compared with DGC, suggesting that avoidance of centrifugation may preserve a greater proportion of viable spermatozoa. Mechanical stress during centrifugation has been associated with sperm loss and increased reactive oxygen species generation, providing a plausible biological explanation for this finding (12, 26).

Although both techniques effectively reduced SDF, the higher motile yield achieved with Harvester may provide a practical laboratory advantage. Similar reductions in DNA fragmentation after microfluidic sperm selection have been demonstrated in controlled laboratory settings (10, 13, 27). Moreover, previous comparative studies indicate that microfluidic selection may reduce oxidative stress markers compared with centrifugation-based methods, and enhanced recovery without centrifugation has also been reported in migration-based platforms, supporting the biological plausibility of our findings (13, 27-29).

### Clinical Relevance

Both DGC and Harvester appear effective in reducing baseline DNA fragmentation at the laboratory level. The higher motile sperm recovery observed with Harvester may represent a practical advantage, particularly in cases with low sperm counts or compromised semen parameters. However, no clinical outcomes were evaluated in this pilot study. Therefore, the extent to which these laboratory differences translate into improved fertilization, embryo development, or live birth rates remains unknown and requires investigation in adequately powered clinical studies.

### Strengths and limitations

The strengths of this study include its within-subject design, minimizing inter-individual variability, and the use of a validated SCD methodology with both manual and CASA-based assessment. The incorporation of agreement analysis and bootstrap confidence intervals adds methodological robustness, particularly in the context of a limited sample size.

This study also has limitations. The small sample size restricts the precision of estimates and limits generalizability. Only a subset of samples underwent SDF assessment, and oxidative stress markers were not directly measured, precluding mechanistic conclusions. In addition, as a pilot study, no formal sample size calculation was performed, and findings should be interpreted as hypothesis-generating.

### Future Directions

Larger, prospective studies are needed to confirm these findings and determine whether non-centrifugal sperm selection translates into improved reproductive outcomes. Future research should incorporate clinical endpoints, oxidative stress biomarkers, and functional sperm assays to better elucidate the mechanisms underlying observed differences. Evaluation of reproducibility across laboratories and cost-effectiveness will also be important for broader clinical implementation.

## CONCLUSIONS

Both DGC and SwimCount Harvester™ significantly reduce sperm DNA fragmentation compared with raw semen, with no significant difference between methods. The Harvester system yields higher total motile sperm recovery and demonstrates excellent agreement between manual and CASA-based SDF assessment. These findings support the potential of migration-based, non-centrifugal sperm selection as an alternative to conventional centrifugation, particularly in cases where maximizing motile sperm recovery is desirable. Further studies are required to establish clinical relevance.

## Data Availability

Trial data will be made available on reasonable request to the corresponding author.
